# Phage-layer interferometry: a companion diagnostic for phage therapy and a bacterial testing platform

**DOI:** 10.1038/s41598-024-55776-1

**Published:** 2024-03-12

**Authors:** Patrick Needham, Richard C. Page, Kevin Yehl

**Affiliations:** Department of Chemistry and Biochemistry, Miami University, Oxford, 45056 USA

**Keywords:** Bacterial infection, Biosensors, Biosensors

## Abstract

The continuing and rapid emergence of antibiotic resistance (AMR) calls for innovations in antimicrobial therapies. A promising, ‘re-emerging’ approach is the application of bacteriophage viruses to selectively infect and kill pathogenic bacteria, referred to as phage therapy. In practice, phage therapy is personalized and requires companion diagnostics to identify efficacious phages, which are then formulated into a therapeutic cocktail. The predominant means for phage screening involves optical-based assays, but these methods cannot be carried out in complex media, such as colored solutions, inhomogeneous mixtures, or high-viscosity samples, which are often conditions encountered in vivo. Moreover, these assays cannot distinguish phage binding and lysis parameters, which are important for standardizing phage cocktail formulation. To address these challenges, we developed Phage-layer Interferometry (PLI) as a companion diagnostic. Herein, PLI is assessed as a quantitative phage screening method and prototyped as a bacterial detection platform. Importantly, PLI is amenable to automation and is functional in complex, opaque media, such as baby formula. Due to these newfound capabilities, we foresee immediate and broad impact of PLI for combating AMR and protecting against foodborne illnesses.

## Introduction

Antibiotic resistance is an urgent public health threat, resulting in ~5 million deaths annually worldwide^1^. In the United States alone there are ~3 million infections a year, causing 35,000 deaths^2^. The incidence of antibiotic-resistant infections is growing due to a complex combination of factors, over prescription and misuse of antibiotics, industrial-scale application of antibiotics in livestock, and globalization increasing the spread of antibiotic-resistant pathogens^3,4^. Furthermore, COVID-19 is expected to exacerbate antibiotic resistance further because of the increased usage of antibiotics for treating bacterial secondary infections stemming from COVID-19^5,6^. The current standard of care for treating antibiotic-resistant infections is to treat with other types of antibiotics. Typically, these classes of antibiotics are administered intravenously and are more toxic, which is problematic for patients in poor health and who are typically the most vulnerable to bacterial infections. In cases where the last line of defense antibiotic fails, the patient must undergo surgical removal of infection, live with recurring infections, or even succumb to infection^7,8^. However, bacteriophage (phage) therapy offers a promising solution, which is the application of viruses to treat infection^9–11^.

Phage therapy has a deep history dating back over a century and is common practice in Eastern Europe. It is also practiced in the United States for compassionate care use^12,13^. A major advantage of phage therapy is that bacteriophages are insensitive to common antibiotic resistance mechanisms, so they can be used to treat multidrug-resistant infections^14^. In addition, bacteriophages have narrow host range, so are targeted to a particular bacterial species or even strain, thus precludes dysbiosis^14–16^. However, a narrow host range requires administering phage therapy as a mixture of different phage types to treat polyclonal infections or to minimize resistance development^15,16^. This mixture can be formulated as a ‘universal cocktail’, which is a predefined mixture of phages targeting a broad collection of pathogenic isolates, or as a ‘personalized cocktail’, a mixture of phages tailored to the infective pathogen(s)^15,17–19^. Though the ‘universal cocktail’ approach is more amenable to current regulatory practices and for scaling production, the ‘active phages’ specific to the infective pathogen are diluted with non-efficacious phages^20^. Great efforts are being made to broaden phage host range through synthetic biology to minimize this ‘dilution effect’ and to simplify production, though these efforts are ongoing^15,16,21–27^.

Comparatively, personalized phage therapies have increased efficacy because all phages are active against the infectious pathogen^17^. Additionally, a personalized phage cocktail minimizes the number of phage types in the mixture, thus reducing potential immunogenic side effects. As such, the personalized approach is mostly practiced in the US when using phage therapy^17,20,28–30^. However, this strategy requires identifying phages able to infect the pathogen-of-interest. Traditionally, this is achieved through the classical microbiology double-layer agar (DLA) assay, where phage suspensions are spotted onto a bacterial agar ‘lawn’ at varying dilutions. Upon phage replication and lysis, a translucent spot is formed and counted to quantify the number of infective viral particles as plaque-forming units (PFU)^31^. Though effective and having been performed for many decades, this classical microbiology assay is labor-intensive and not amenable to automation for high-throughput screening. Additionally, it is time-consuming, requiring overnight cultures of bacteria and phage growth, and cannot capture phage-host dynamics. Furthermore, DLA is limited to agar plates, which does not recapitulate infection conditions. Thus, there is a major need to develop companion diagnostics that quickly identify efficacious phages; are amenable to automation and scale-up; and can quantify phage virulence to maximize the effectiveness of phage therapies.

Towards this goal, many methods are being developed. Storms et al. and Konopacki et al. developed methods to quantify a phage-of-interest’s ‘virulence index’ or ‘phage score’, respectively, by measuring bacterial killing dynamics at varying multiplicities of infection (MOIs), which is the ratio of phage to bacteria^32,33^. This method is high-throughput and can quantitatively assess phage virulence to formulate phage cocktails in a more standardized way. However, this approach uses optical density as a readout for bacterial viability, in which bacterial cellular debris from lysis may obscure results and underestimate phage lytic activity. This can be overcome by using the OmniLog^TM^ system, which uses redox chemistry to monitor bacterial metabolic activity by including a tetrazolium dye at 1% (v/v) in the growth medium^34^. During bacterial growth, respiration reduces the tetrazolium dye and produces a color change, thus phage mediated lysis results in a decreased color change compared to bacteria grown in the absence of phage. The OmniLog^TM^ system is extremely high-throughput, having the capacity to monitor 50 microtiter plates at a time, and can carry out 4800 phage assays simultaneously. Importantly, bacterial debris does not interfere with the signal readout. Though both these methods are high-throughput, they use a turn-off signal to assess phage virulence and host range^34^. In principle, this limits sensitivity. Very recently, Edigo et al. developed a fluorescent turn-on assay by adding Sytox green fluorescent dye to the growth medium, which is a membrane-impermeable nucleic acid dye that fluoresces when bound to DNA. Phage-mediated lysis results in the release of bacterial DNA and an increase in fluorescent signal^35^. Together, these approaches are great advances towards standardizing quantification of phage virulence and measuring phage host range in a high-throughput manner. However, each method requires phage plaquing to determine MOI for virulence quantification, which is laborious. Most importantly, these methods cannot be carried out in complex media, such as colored or high-viscosity solutions or inhomogeneous mixtures, which are often conditions encountered in vivo. To address this technology gap, we hypothesized that biolayer interferometry (BLI) can be used as a complementary method to quantify phage-host dynamics that is amenable to automation, high-throughput, and functional in complex media.

**Figure 1:**
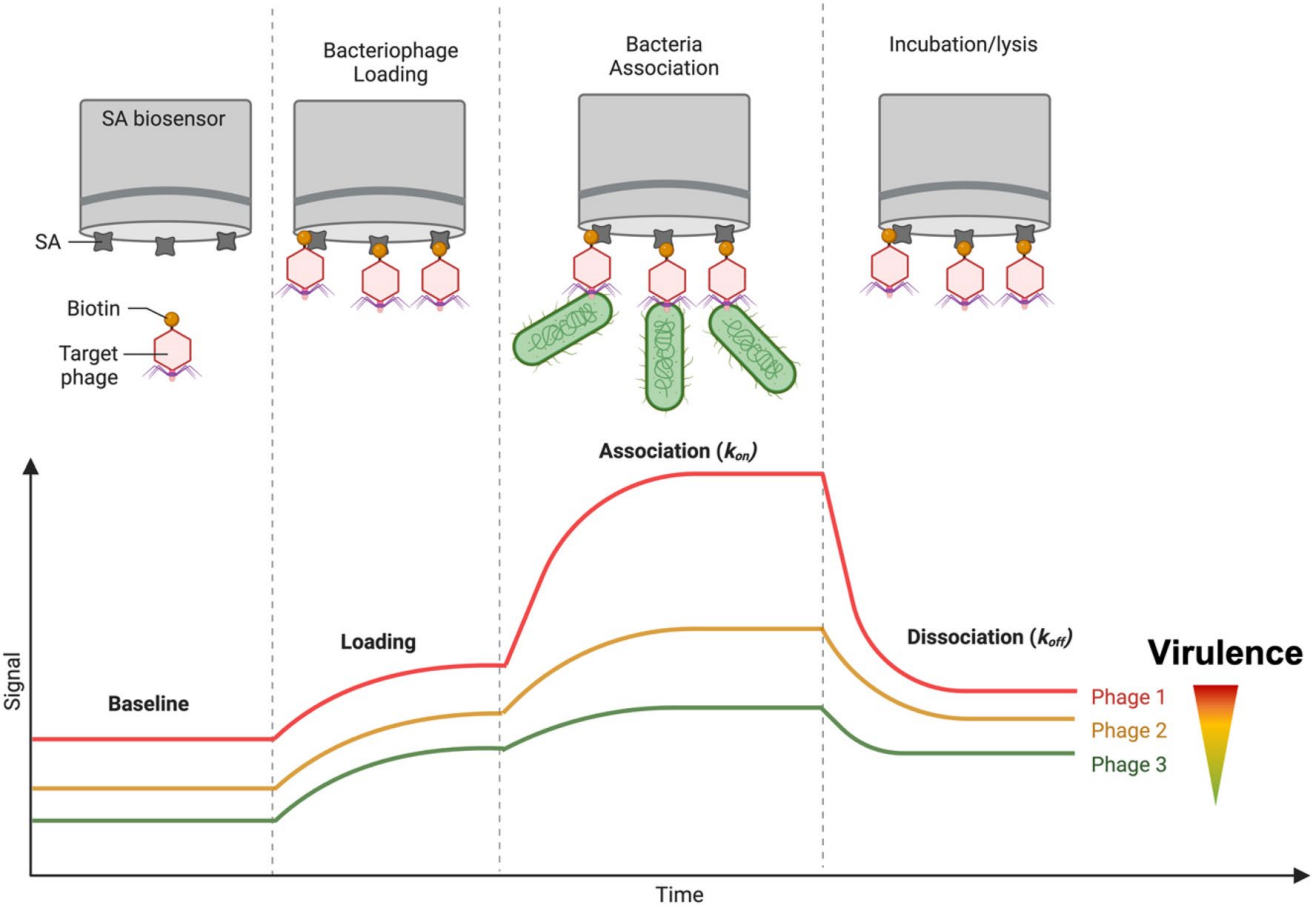
Overview of the PLI concept and proposed real-time signal readout for bacterial binding and lysis.

BLI is a real-time surface-based analytical technique that is traditionally used for quantifying biomolecular interactions through monitoring convergent and divergent interactions of white light reflected from a fiber-optic biosensor surface^36^. An increased number of biomolecules bound to the sensor surface results in a red shift in reflected light. This is converted to a ‘binding’ signal and plotted as a function of time, thus producing a sensorgram, from which kinetic parameters can be derived^37^. Consequently, BLI-based diagnostics can distinguish phage binding from lysis, unlike current methods. Importantly, BLI can operate in complex media, including inhomogeneous mixtures and opaque and high-viscosity solutions^38–42^. We believe that these capabilities provide another dimension for studying phage-host (bacterial or immunological) dynamics that is not possible with currently available methods. To highlight these capabilities, we functionalized and characterized T7 phage biosensors and studied phage-host dynamics on a collection of 30 *E. coli* isolates. We refer to this method as phage-layer interferometry (PLI) (Fig. 1). Furthermore, we demonstrate that PLI has broad utility and can be used to detect bacterial contamination of baby formula in real-time, thus exhibiting the ability to study phage-host dynamics in a complex media.

## Results

### Phage and sensor functionalization and characterization

**Figure 2:**
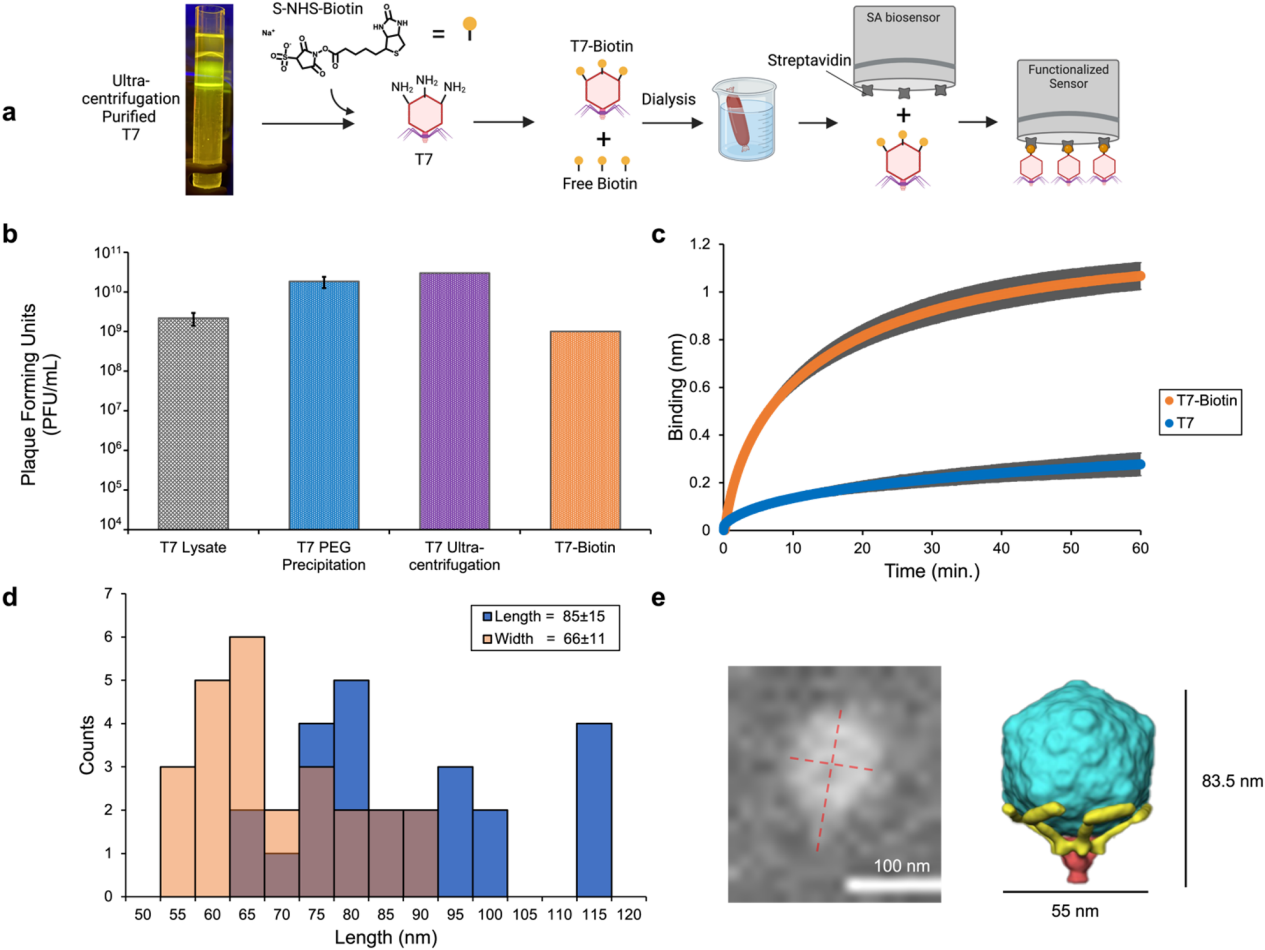
Phage and Sensor Functionalization. (**a**) Schematic depicting the steps to functionalize T7 with biotin-NHS and then load onto a streptavidin (SA) biosensor. (**b**) Average T7 activity throughout the functionalization process was determined by plaque assays and are shown as the mean of 3 replicates with error bars representing the standard deviation. (**c**) Overlayed sensorgrams comparing specific binding of T7-bio to nonspecific binding of wild-type T7 to SA biosensors. (**d**) Histogram summarizing phage particle diameters (short and long axes) functionalized to the SA biosensor obtained from SEM analysis. *n* = 25 phage particles compiled in Supplementary Fig. S2. (**e**) A representative SEM image of T7-bio loaded onto the SA biosensor surface compared to a 3D model of T7 rendered from cryo–electron tomography (cryo-ET)^45^.

T7 was used as the phage biosensor to prototype PLI because it has high therapeutic potential. It is active in vivo, can be engineered to expand host range to target an array of pathogens, and can be used as a phagemid delivery system^15,16,21,43^. To synthesize phage-functionalized BLI sensors, biotin-streptavidin bioconjugation was used. Briefly, T7 phage was biotinylated using a sulfo-NHS-biotin heterobifunctional linker and then immobilized onto a streptavidin (SA) coated BLI-biosensor (Octet® SA biosensor). Since amine reactive molecules can quench the NHS reaction and are present in crude phage lysates, T7 lysates were PEG precipitated, purified via ultracentrifugation, and dialyzed prior to NHS conjugation (Fig. 2a). Specifically, T7 lysates were grown in eight 100 mL cultures using *BW25113* as the production strain. The resulting lysates were concentrated 10× via PEG precipitation and purified by cesium chloride (CsCl) density gradient ultracentrifugation to remove smaller amine reactive molecules. CsCl was then buffer exchanged with PBS for the sulfo-NHS reaction. Following biotin-NHS labeling, the biotinylated-phage (T7-bio) was dialyzed in PBS to remove excess biotin^44^.

After each step, phage activity was assessed via plaquing to quantify the remaining number of infective particles (Fig. 2b). Importantly, centrifugal forces, biotinylation, and dialysis did not result in significant loss in the number of infective particles. To maximize T7 loading onto the sensor, T7-bio was titrated onto the sensor surface in a series of four incubation steps, where the concentration of T7 increased by 10× and ranged from 2 × 10^5^ to 3 × 10^8^ PFU/mL in 200 μL (Supplementary Fig. S1). It was proposed that this would provide a saturated sensor surface and an increase in PLI signal. The optimal T7-bio loading concentration was determined to be 2 × 10^7^ PFU/mL because it produced the largest signal response (Supplementary Fig. S1, gray). Optimized loading of the phage sample onto the SA biosensor is shown in Fig. 2c, where wild-type T7 (non-biotinylated) (2 × 10^10^ PFU/mL) was used as a control to differentiate non-specific binding. It is important to note that wild-type T7 is a thousand times more concentrated compared to T7-bio, yet produces a significantly lower binding signal, thus showing selective T7-bio sensor loading.

Next, scanning electron microscopy (SEM) was performed on the sensor surface to confirm T7 functionalization. Figure 2d shows a histogram summarizing single particle analysis (Supplementary Fig. S2) of the immobilized T7 particles, displaying an average length and diameter of 85 ± 15 nm and 66 ± 11 nm, respectively. This agrees well with the dimensions calculated from a 3D cryo-electron tomography derived model from Hu et al., having dimensions of ~ 84 × 53 nm^45^. Figure 2e shows a representative SEM image of an immobilized T7 particle compared to the cryo-electron tomographic 3D model^45^.

Since the fiber optic has a large surface area that is not part of the sensor surface and where phage and bacteria can nonspecifically bind, the sensor was washed to remove any nonspecific bound T7 and bacteria (Supplementary Fig. S3). The wash steps were optimized by testing various wash solutions and varying the number of washes. The solutions investigated were PBS, PBS tween-20 (PBS-T, 0.1% v/v), and LB. These solutions were examined by monitoring the loss in PLI signal over time and measuring plaquing of each wash solution to quantify the number of nonspecifically bound phages. The wash protocol was also investigated for removing nonspecifically bound *BW25113* from the biosensors following bacterial association by similarly monitoring the loss in PLI signal and enumerating the number of nonspecifically bound bacteria in the wash buffers. Representative sensorgrams showing overlays of each wash step for PBS-T are shown in Supplementary Fig. S3, along with corresponding plaquing and bacterial titers. Comparisons of all the buffers are shown in Supplementary Fig. S3 and show that PBS-T was the best buffer for removing nonspecifically bound T7 and *BW25113*. All buffers were efficient at removing nonspecifically bound phage, where PBS-T washed away nonspecifically bound bacteria to below detectable levels (< 10^3^ CFU/mL).

### Detecting bacterial binding and lysis

**Figure 3:**
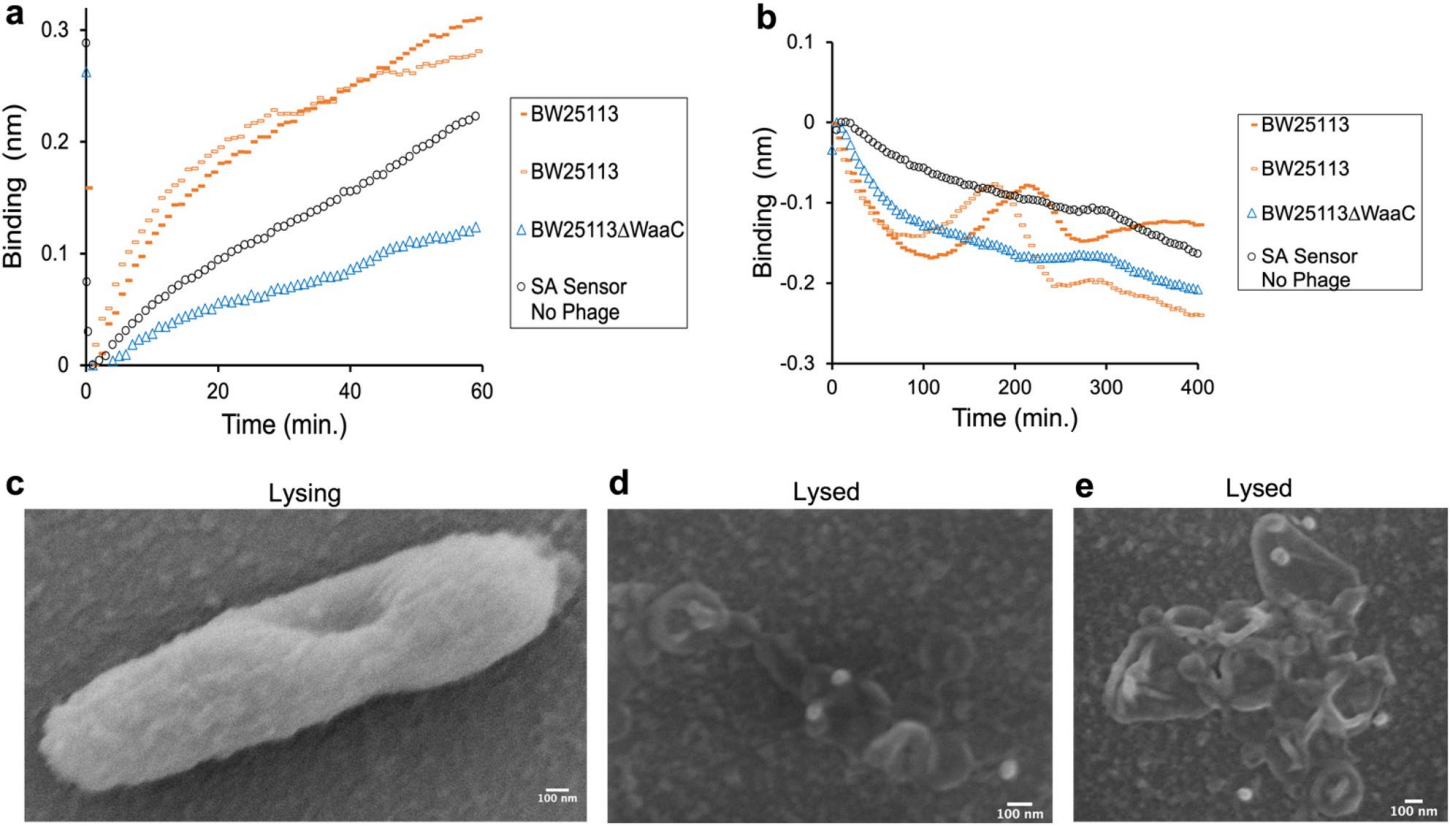
Real-time PLI analysis of bacterial binding and lysis along with SEM validation. (**a**) Overlayed sensorgrams showing bacterial binding for *BW25113* and T7 resistant mutant (*BW25113ΔwaaCΔtrxA*) to the T7-bio functionalized PLI sensor. Two independent replicate trials are shown for *BW25113* (*BW25113,* orange dashes; *BW25113ΔwaaCΔtrxA*, blue triangles; and SA Sensor No T7-bio, black circles)*.* (**b**) Overlayed sensorgrams showing bacterial lysis for *BW25113* (orange dashes) and no lysis for *BW25113ΔwaaCΔtrxA* (blue triangles). SA Sensor no T7-bio, shows bacterial growth*.* (**c–e**) SEM images of the biosensor surface confirming bacterial binding and lysis.

To demonstrate the utility of PLI for measuring phage host range and quantifying phage infection parameters, such as host binding and latency period, phage-host dynamics were studied by submerging T7-sensors in bacterial broth cultures and measuring PLI signal over time. Both *BW25113,* a known phage-sensitive strain, and *BW25113ΔwaaCΔtrxA*, an engineered T7 resistant strain, were initially tested^21^. *BW25113ΔwaaCΔtrxA* has two mutations that make it T7 resistant by blocking T7 binding and inhibiting T7 replication, respectively^21^. Specifically, *waaC* is part of the *waa* gene cluster and encodes for an enzyme involved in LPS core biosynthesis^46^. The *ΔwaaC* gene deletion results in a truncated LPS lacking nearly all of the outer core, including the glucose moiety that T7 uses as a receptor^15,47^. *trxA* encodes for thioredoxin 1, a processivity factor for T7 RNA polymerase and a known essential host gene for T7 replication^48^. PLI sensorgrams showing the dynamics of these two strains were overlaid to compare bacterial association (Fig. 3a) and phage lysis, or lack thereof (Fig. 3b). A representative sensorgram showing all the steps involved in PLI: sensor loading, washing, bacterial association and lysis is shown in Supplementary Fig. S4.

As expected, *BW25113* had stronger association to the T7-PLI sensor (Fig. 3a, orange dashes) compared to *BW25113ΔwaaCΔtrxA* (Fig. 3a, blue triangles), as indicated by a 3.4x larger slope and higher binding signal after 60 min. The bacterial binding was determined to be specific to the T7 biosensor by comparing sensorgrams to SA sensor only, i.e., sensor with no phage, that were mixed with *BW25113* (Fig. 3a, black circles). Two independent trials of *BW25113* dynamics were tested to evaluate PLI reproducibility, which showed an average standard deviation of ±0.088 nm between the two runs. Importantly, this highlights the capability of PLI to compare phage binding parameters when investigating phage host range, as T7 has a much stronger association and faster binding kinetics to *BW25113* compared to *BW25113ΔwaaCΔtrxA*.

After bacterial association, sensors were washed and incubated in LB to measure lysis dynamics (Fig. 3b). A slight decrease in PLI signal was initially observed for both *BW25113* and *BW25113ΔwaaCΔtrxA*, which can be attributed to bacterial dissociation. Interestingly, after ~120 min, a sharp increase in signal was observed for *BW25113*. We originally hypothesized that this was due to bacterial replication. This signal increase could also be due to bacteria changing morphology, such as bacterial swelling induced by phage infection. The latter is corroborated by recent reports of single-molecule fluorescence studies monitoring T7-bacterial dynamics, where an increase in surface roughness and 200–500 nm bleb formation were observed in early T7 infection^49,50^. Following this phase, only *BW25113* showed a rapid decrease in PLI signal, indicative of sudden lysis and diffusion of bacterial debris away from the sensor surface. As expected, *BW25113ΔwaaCΔtrxA* did not produce a ‘lysis’ signal, which is consistent with reports that *ΔtrxA* severely inhibits T7 propagation^21,46,51^. SA sensor only, no T7, only showed bacterial dissociation of non-specifically bound *BW25113* (Fig. 3b, black circles).

Since single infection cycles are observed in PLI, the latency period can easily be deduced, i.e., the time taken by a phage particle to reproduce inside an infected host cell. To quantify the latency period, we took the first derivative of the sensorgram and found the time point when the rate of change, *d*_*binding*_/*d*_*t*_, is equal to zero. Our results show that T7 has a latency time of ~193 ± 26 min at room temperature. The slight difference between runs can be attributed to the small temperature variations between experiments, which were run on different days at room temperature (~23 °C). We anticipate that running PLI under temperature control at 37 °C will reduce error and also result in much faster lysis times, ultimately decreasing the overall time for characterizing phage host range and virulence. Traditionally, the latency period is measured by single-growth kinetics, where bacteria are infected with phage at a high MOI (>1), and bacterial growth is observed. Unlike PLI, this approach requires plaquing to determine MOI, which is labor-intensive. Additionally, the traditional approach is very sensitive to experimental parameters, such as bacterial growth phase.

To confirm bacterial lysis, SEM was used to visualize bacterial debris on the sensor surface. T7-PLI sensors were incubated with *BW25113* for bacterial association and then washed and incubated in LB until lysis signal was observed. After ~12 hr., sensors were removed from LB and submerged in paraformaldehyde fixative to preserve the biological structure. Representative images showing bacterial lysis and lysed product are shown in Fig. 3c-e. Importantly, lysis debris is overlaid with immobilized T7-biosensors (white colored particles), thus confirming phage sensor-induced lysis.

**Figure 4:**
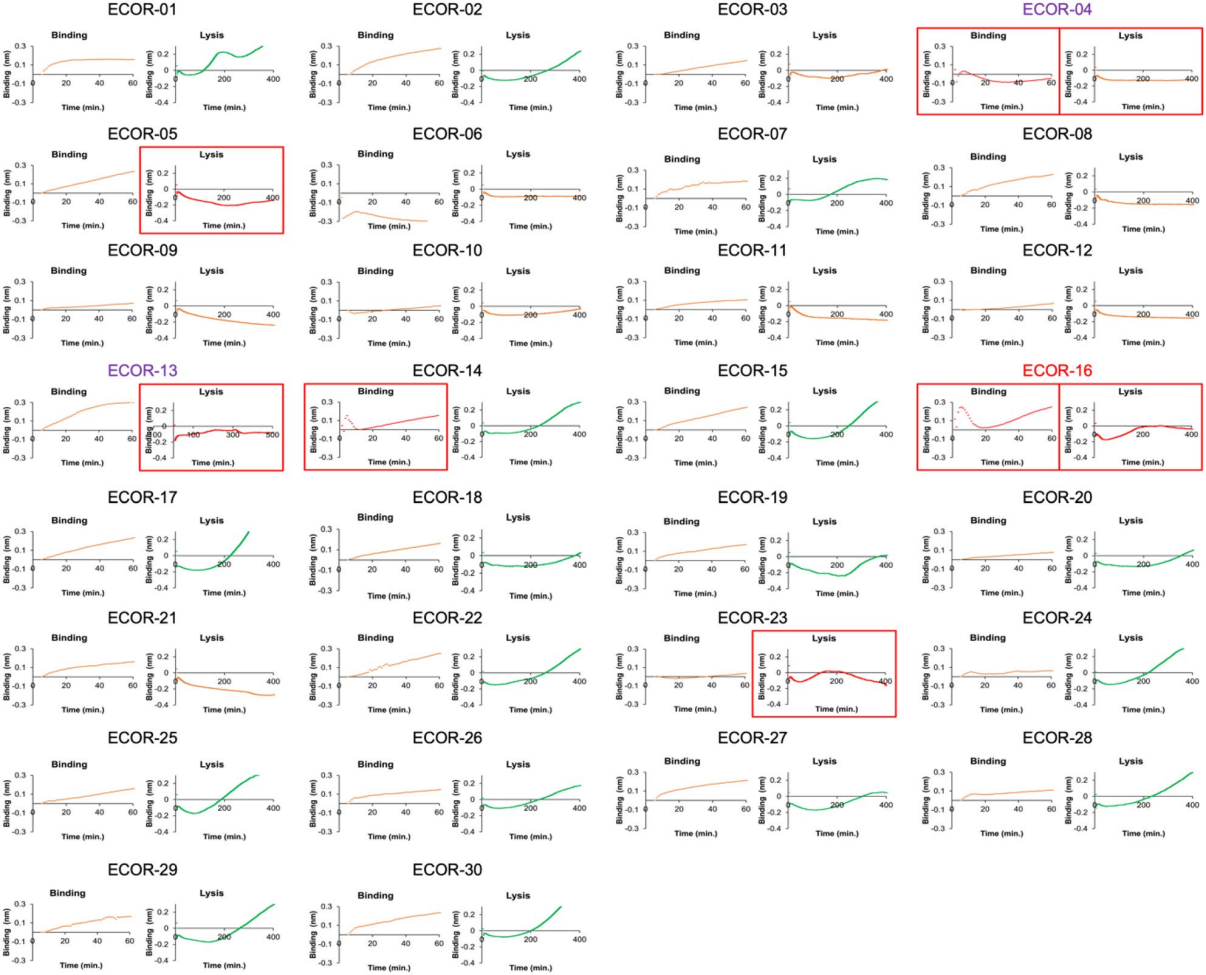
Screening T7 host range using PLI. A summary of binding and lysis sensorgrams for the 30 strains of the ECOR collection. Red boxes and red lines indicate lysis; green lines indicate growth; purple font indicates strains that were identified as sensitive from the DLA and kinetic growth assay; red font indicates strains identified as sensitive from DLA. Objective criteria to determine PLI-based phage sensitivity are summarized in Supplementary Note I in supplementary information.

Next, to test whether PLI can be used to screen phage host range to identify phage sensitive and insensitive strains, T7 host range was screened using part of the ECOR collection. The ECOR collection comprises *E. coli* strains from geographical locations all around the world and isolated from a variety of hosts, including animals and humans^52^. To benchmark PLI, traditional phage screening methods, such as DLA and kinetic growth assays, were carried out and are summarized in Supplementary Figs. S5 and S6, respectively. DLA screening identified three sensitive strains: ECOR-04, ECOR-13, and ECOR-16, which are highlighted by red boxes (Supplementary Fig. S5), where the kinetic growth assay only showed ECOR-04 and ECOR-13 as being sensitive, highlighted by blue boxes (Supplementary Fig. S6). A summary of all PLI binding and lysis sensorgrams for screening the ECOR collection are shown in Fig. 4 and provide a wealth of information, showing varying binding affinities, unique lysis signals (red lines), and insensitivity/resistance (green lines). Objective criteria for determining sensitivity are summarized in the supplemental under Supplementary Note I. Strains that show lysis are highlighted by red boxes and red lines. Briefly, a rapid binding signal followed by a sudden drop is indicative of strong binding and sudden lysis, which is observed for ECOR-04, ECOR-14, and ECOR16. Also, strains that produced a negative slope during the lysis step and had a poor R^2^ fit, accounting for increasing signal due to morphological changes and then lysis, were also classified as sensitive strains, which were ECOR-04, ECOR-05, ECOR-13, ECOR-16, and ECOR-23. Strains that had weak binding also showed a negative slope during the lysis step due to bacterial disassociation, but had a strong linear fit (R^2^ > 0.5). This enabled differentiating insensitive strains from sensitive strains that lysed. For comparison, ECOR strains that were identified as sensitive from both DLA and kinetic growth assays are indicated by purple font, where strains identified as sensitive from DLA only are indicated by red font. Interestingly, lysis was observed for all these strains using PLI. In fact, originally ECOR-16 was not identified as sensitive in the DLA assay, but upon observing lysis signal in the PLI sensorgram, the DLA petri dish was re-analyzed and showed small plaques at the most concentrated dilution. PLI also identified other potentially sensitive strains: ECOR-05, ECOR-14 and ECOR-23, and possible explanations for why T7 sensitivity was not detected in the DLA- or kinetic growth curve assays. For example, ECOR-14 showed lysis during the binding step (i.e., a rapid increase in signal followed by sudden decrease in signal), but then shows regrowth (green line), indicating resistance. ECOR-23 shows a clear lysis signal during the lysis step, but has very weak binding, so if binding can be improved, T7 potentially can infect more effectively. ECOR-05 shows medium to strong binding and a lysis signal. Together, these results highlight PLI’s capability to measure phage host range and readily compare phage infection parameters, such as host binding kinetics and latency period, thus enabling a more standardized process to screen phage candidates.

### Measuring label free phage dynamics

**Figure 5:**
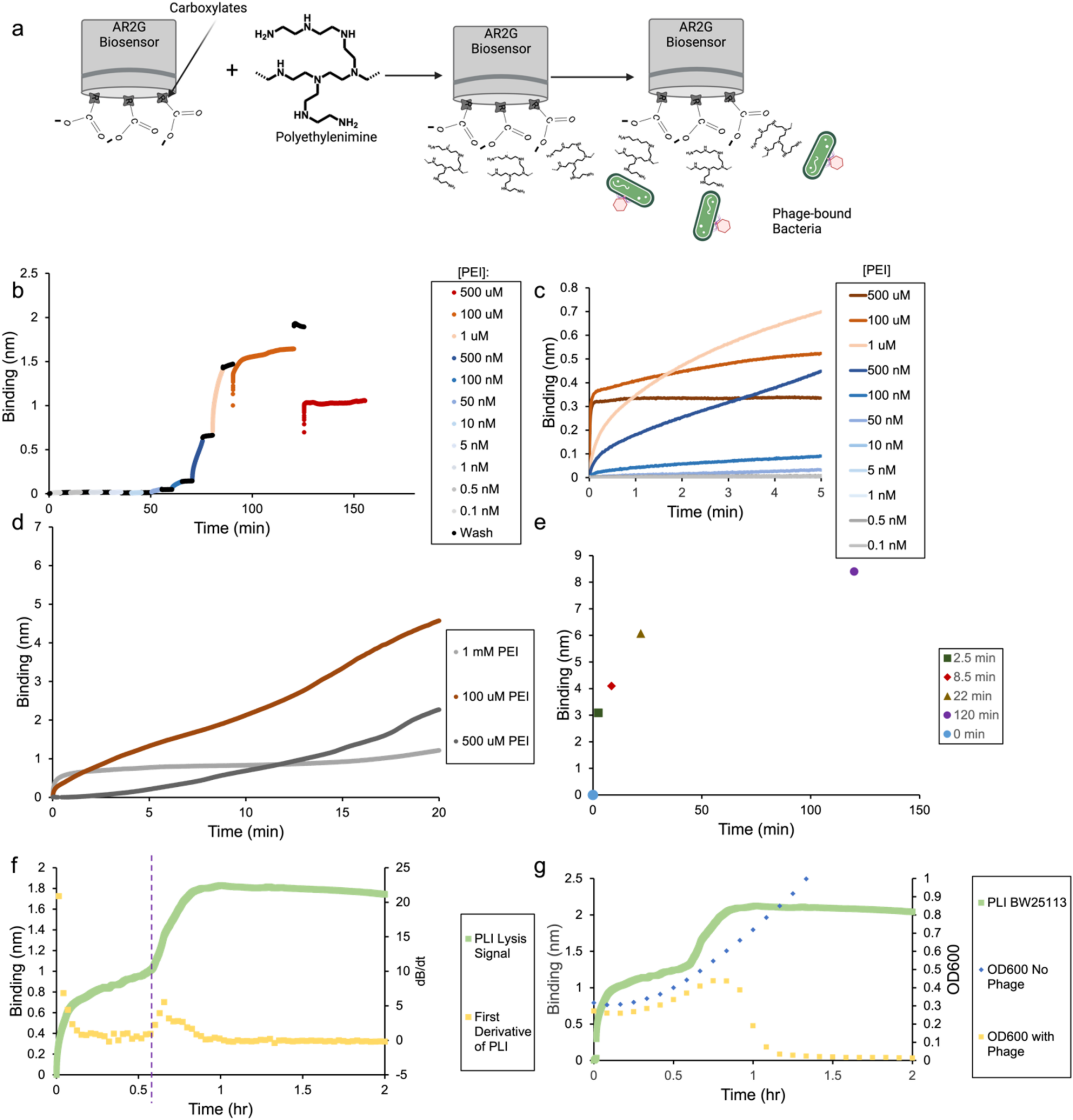
PEI sensor functionalization and PLI dynamics of label-free phages. (**a**) Schematic depicting the sensor loading and bacterial capture procedure. (**b**) Sensorgram showing titration of PEI loading onto AR2G sensors at varying concentrations (pM, gray; nM, blue; and μM, red). The loading is indicated by color curves and the wash steps are indicated by black curves. (**c**) Overlayed sensorgrams of the PEI titration showing only the PEI loading step. (**d**) Overlayed sensorgrams showing *BW25113* association to AR2G sensors loaded with varying concentrations of PEI (500 μM PEI, dark gray; 100 μM PEI, red; and 1 mM PEI light gray). (**e**) A plot summarizing the maximum binding signal for *BW25113* association to PEI functionalized sensors (100 μM) at varying time points compiled from five independent experiments. (**f**) PLI sensorgram (green squares) showing bacterial binding and lysis of *BW25113* by label free T7 with overlayed 1st derivative curve (yellow squares). (**g**) Overlayed sensorgrams showing bacterial lysis for *BW25113* (green squares), compared to spectroscopic *BW25113* growth curves with and without T7 (yellow squares and blue diamonds, respectively).

Since the current T7-bio purification procedure requires ultracentrifugation, which is not compatible with all phage types, we were curious whether PLI can be used to measure phage dynamics of non-biotinylated phages^53^. To test this, we functionalized amine reactive 2nd generation sensors (Octet®AR2G), which has a carboxylated surface and is negatively charged, with positively charged polethylenimine (PEI) polymer (MW: 10,000 g/mol), to capture *BW25113* bound with phage (Fig. 5a), and then monitor PLI dynamics. *BW25113* binds to the PEI functionalized sensor because *E. coli* has a net negative surface charge and associates with positively charged surfaces through ionic interactions^54^. We chose to functionalize the sensor with PEI rather than testing positively charged sensors directly (Octet®APS) because the surface charge can be tuned depending on the amount of PEI used for sensor loading. This is important as a surface with too high a zeta potential is antimicrobial and will result in bacterial lysis^55,56^. To determine the optimal loading of PEI and whether PEI binds irreversibly to Octet®AR2G sensors, a titration of varying concentrations of PEI was tested, spanning 0.1 nM to 500 μM PEI, and having wash steps in between (Fig. 5b). Since no loss in signal was observed during the wash steps, PEI binds irreversibly to the Octet®AR2G surface and is very stable. The resulting sensorgrams of PEI loading were overlaid to better compare the amount of PEI functionalization (Fig. 5c). Interestingly, the change in binding signal was highest at 10 μM PEI compared to 100 μM and 500 μM PEI. We speculate that this is due to PEI loading being saturated at this concentration. The optimal PEI loading for bacterial capture was empirically determined by carrying out bacterial binding assays and monitoring the PLI signal over time (Fig. 5d). The optimal PEI loading concentration was determined to be 100 μM, as these sensors had the fastest bacterial capture kinetics and produced the largest binding signal, where higher concentrations of PEI resulted in slower binding kinetics and lower signal (Fig. 5d), most likely due to bacterial lysis. To assess the assay’s reproducibility, binding signals for bacterial association were measured from five independent experiments at varying time points and compiled into a single plot (Fig. 5e). This analysis showed that the bacterial capture is highly reproducible, showing saturation in bacterial binding after ~120 min.

Next, phage lysis dynamics were studied by mixing wild-type T7 (unlabeled) with *BW25113* for 15 min at an MOI of 2, washing with PBS (4× times), and then loading onto the PEI coated sensor to observe binding and lysis dynamics at 37 °C. In the first 5 minutes, a rapid increase in binding signal was observed, which is attributed to binding of small negatively charged biomolecules as bacteria have much slower diffusion comparatively. This phase is followed by a slow and steady increase in bacterial association, which is followed by a sudden increase in PLI signal after ~40 min. We hypothesize that this rapid increase in signal is due to phage-induced lysis, releasing negatively charged biomolecules that then bind to the positively charged sensor surface. This results in a ‘turn on’ lysis signal, compared to the ‘turn-off’ lysis signal previously observed with the SA sensors. The lysis time was quantitatively assessed by taking the first derivative of the PLI curve, and finding the minima where the slope begins to rapidly increase (Fig. 5f). The lysis time was measured to be ~37 minutes and is indicated by a purple dotted line. The phage lysis time is much faster at 37 °C compared to room temperature, which was previously measured to be 193 min (Fig. 3b). To corroborate that the rapid signal increase is indeed due to phage induced lysis, bacterial growth was monitored spectroscopically using a plate reader, which was overlaid with the sensorgram (Fig. 5g, dotted lines). The rapid loss in optical density (OD_600_) correlates with the increase in PLI signal, thus confirming that the rapid signal increase is due to lysis. Together, these results show that PLI can be used to monitor phage-host dynamics of label free phages and not require biotin functionalization.

### Using PLI to detect bacterial contamination of baby formula

**Figure 6:**
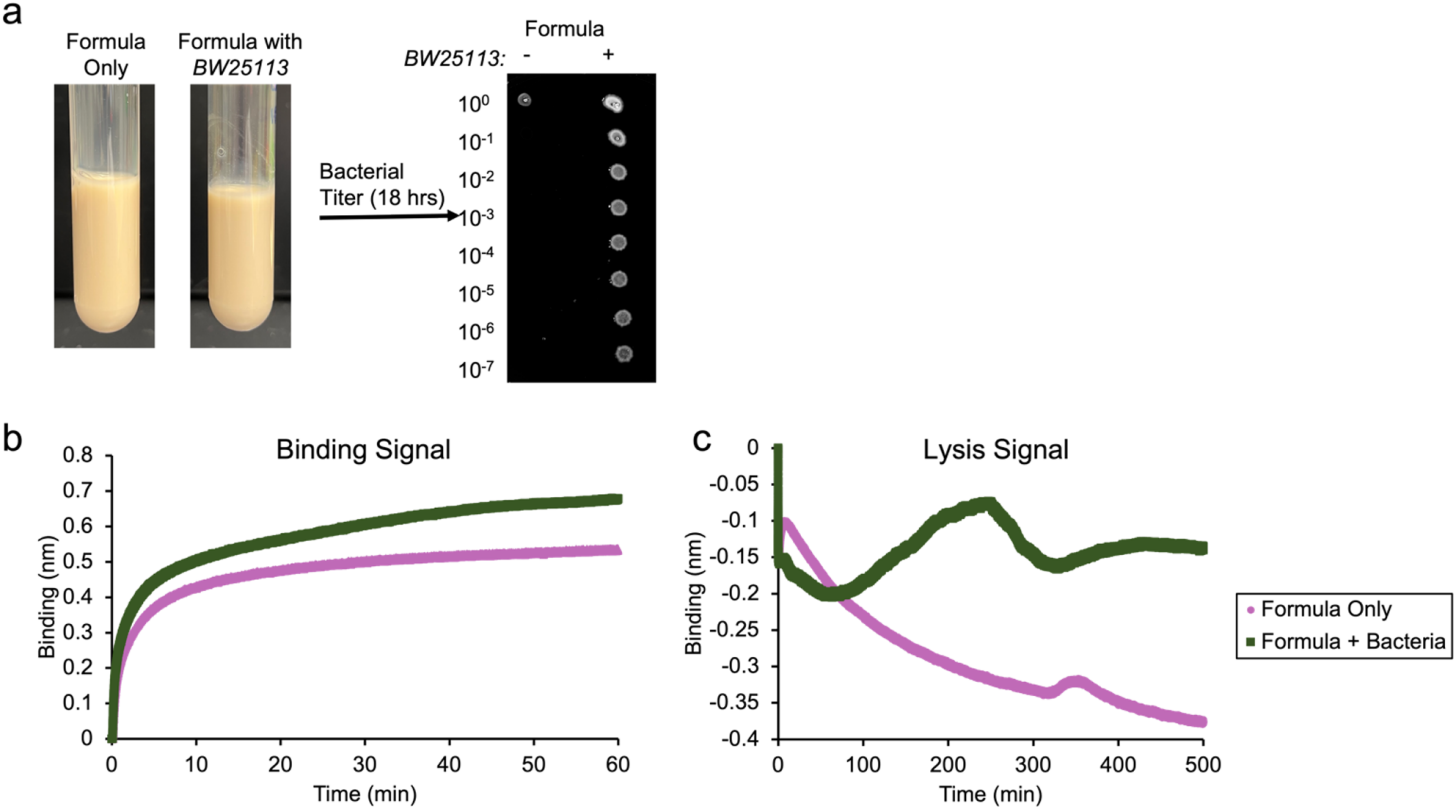
PLI detection of contaminated baby formula. (**a**) Images showing formula only (left) and formula spiked with *BW25113* (right) with corresponding bacterial enumeration assays. (**b, c**) Overlayed sensorgrams showing binding (**b**) and lysis (**c**) signal of contaminated formula (green squares) compared to noncontaminated formula (pink circles).

Since inherent to any phage companion diagnostic is the ability to detect bacteria and because BLI has the capability to operate in complex media, we hypothesized that PLI can be used to detect bacterial contamination of baby formula. Baby formula was chosen as the target medium because recently in the United States contaminated baby formula resulted in a nationwide shortage causing a public health crisis^57^. However, baby formula is opaque and difficult to analyze through traditional spectroscopic methods. Figure 6a shows a picture of baby formula and contaminated baby formula with corresponding bacterial enumeration assays. To test whether PLI can detect bacterial contamination in real-time, a T7-biosensor was submerged into contaminated baby formula and the resulting sensorgram was compared to formula only control. A significant binding signal is seen in both samples; however, binding is much larger for the contaminated baby formula (Fig. 6b, green squares) compared to formula only (Fig. 6b, pink circles). To confirm that the signal is not a false-positive, sensors were washed to remove any nonspecifically bound material and then incubated in LB to observe lysis dynamics, or lack thereof (Fig. 6c). Similar to the lysis signature observed previously (Fig. 3b), contaminated formula showed (i) an initial decrease in signal, resulting from bacterial dissociation; (ii) followed by an increase in signal; (iii) and then a sudden decrease in signal due to lysis. Moreover, lysis was observed within the same time period as before, ~ 220 min (Fig. 3b), where non-contaminated formula controls only showed a dissociation signal of nonspecifically bound molecules from the sensor surface (Fig. 6c, pink circles). These experiments highlight the capability of PLI to operate in real-time in complex media, and also addresses a major public health problem of detecting foodborne contamination. This assay is currently a targeted assay, meaning knowledge of the specific bacterial contaminate is required. However, sensor arrays can easily be engineered to detect a wide-panel of possible pathogen contaminates. Although, if the bacterial target is unknown, traditional bacterial detection methods, such as cultures, next-generation sequencing, or other molecular-based testing would be more appropriate.

## Discussion

With the growing public health problem of antibiotic resistance and decreasing research into new antibiotics by large pharmaceutical companies, there is a major need to develop alternative antimicrobials. Currently, phage therapy is the only option for treating bacterial infections that are resistant to the last line of defense antibiotics. However, identifying efficacious phages specific to the bacterial pathogen is a major bottleneck that limits the practicality of phage therapy. This is because current methods for phage testing are time- and labor-intensive, which include plaquing and liquid culture assays. The most commonly used phage testing method is DLA, which is not amenable to automation and scale-up. Additionally, it has high user-to-user variability. Therefore, great efforts are underway to develop bioanalytical assays to standardize phage therapy formulation that are amenable to automation^32–35^. However, these methods are predominantly optical-based, involving measuring optical density (OD) or absorption or fluorescence of a reporter dye as a readout for metabolic activity, so are sensitive to optical interferents and are not functional in complex media. Moreover, these methods cannot quantify phage binding kinetics, which is an important parameter that can be used to standardize the formulation of phage therapies.

Being able to study phage-host dynamics in complex media is very important for the future of phage therapy, as nearly all therapeutically relevant biological fluids encountered by phage therapies are complex, meaning colored, highly viscous, or inhomogeneous. For example, blood is colored; synovial fluid, the fluid between the joints, is non-Newtonian, highly viscous, and normally colorless, except can be turbid or colored when there is an infection; sputum, mucus produced from the lower airways, is colored and viscous; and pleural fluid, liquid that is located between the layers of pleura around the lungs, is clear to yellow. All of these biological fluids are associated with septicemia, joint and implant infections, or bacterial pneumonia, respectively. Very little is known about phage-host (bacteria) interactions in these native contexts, and knowing so may give insights for why certain phage therapies succeed, while others fail. However, due to PLI’s capabilities, we believe PLI has significant potential for being able to study phage-bacterial dynamics in this context.

The present work tackles these challenges by using BLI to study phage-host dynamics. BLI is a common biophysical technique for studying biomolecular interactions. Major advantages of BLI are that it is real-time, surface-based, and amenable to automation. As a result, it requires little sample material (< 6 μL) and minimal user input, thus reducing error. Most importantly, unlike SPR, another real-time surface-based analytical technique, BLI does not require flow, so is operable in complex samples, such as high viscosity solutions or inhomogeneous mixtures. For example, BLI assays have been developed to detect analytes in blood, light soy sauce, and even milk^38–42^. In addition, antibodies or phage components, such as receptor binding proteins or lysins, have been used to functionalize BLI biosensors for detecting foodborne pathogens^40,41^. Biosensors functionalized with antibodies specific for *Salmonella enterica* can detect contamination as low as 1.6 × 10^5^ colony-forming units per milliliter (CFU/mL) in less than 300 seconds^41^. Additionally, biosensors functionalized with phage lysin protein have improved limits of detection, being able to detect 13 CFU/mL of *Staphylococcus aureus* in complex media, such as light soy sauce and ice cubes^40^. Whole phage virions have been used as a biosensor for BLI, where binding kinetics of Phage Sf6 to purified membrane proteins was measured^58^. Though BLI assays have been generated for phage and bacterial screening, none of these assays characterize phage-host dynamics, specifically screening for phage host range and measuring phage infectivity parameters, such as binding kinetics or lysis time, which can be used to standardize formulation of phage therapies.

Herein, we developed a BLI-based assay to measure phage-host dynamics, which we refer to as phage layer interferometry (PLI). We show that T7 retains infectivity when functionalized to a BLI biosensor and produces a unique ‘lysis’ signal when the bacterial host is sensitive. In addition, this lysis signature can be used to improve detection of bacterial contamination by being able to distinguish between bacterial binding from non-specific interferent signal. The lysis signature can also be used to distinguish live and dead bacteria. Since PLI is surface-based, single infection cycles are measured. As such, the lysis time can easily be quantified and does not require knowing the MOI. Using PLI, we measured T7 lysis time for *BW25113* to be ~ 193 ± 26 min at room temperature and ~ 37 min at 37 °C. In addition to measuring lysis time, PLI can be used to distinguish host binding parameters. For example, we showed that T7 had faster association and slower dissociation to *BW25113* compared to *BW25113ΔwaaCΔtrxA*, a known T7 insensitive mutant, respectively. Whereas, the traditional method for measuring phage-host binding is extremely labor intensive and involves many steps, that include (i) mixing phage with its bacterial host at a high MOI, (ii) diluting aliquots into fresh media at various time points, (iii) then centrifuging to separate bound phage from unbound phage, and lastly (iv) plaquing the supernatant to measure loss in PFU over time, which is then fit to a decay curve. Additionally, it is difficult to measure binding parameters for bacteriophages that have fast lysis times, where PLI is a real-time measurement and readily can do so.

However, a potential limitation of PLI is that the biotinylation procedure can be laborious. To address this drawback, we showed that phage-bacterial dynamics can be studied using PEI-loaded sensors. This approach can be used to quickly screen newly isolated phages, whereas the biotinylation approach is more amenable for screening phages from known collections. Another potential limitation of PLI is that it only monitors single generation growth kinetics so cannot assess a phage’s ability to suppress the onset of resistance. However, we did notice bacterial strains that lysed, but then regrew on the chip. Importantly, since PLI can operate in complex media, other important phage-host interactions can be quickly screened, such as sensitivity to neutralizing antibodies directly from blood, which is ongoing.

In summary, current methods for phage testing are time and labor-intensive and require large volumes of pathogenic bacterial cultures. Conversely, PLI is straightforward and requires very little bacterial sample. By having a monolayer layer of phage on the sensor surface, single infection cycles are studied. This minimizes assay time and avoids the need for phage plaquing to quantify phage virulence. Additionally, relevant phage infection parameters, such as adsorption rate and latency period, can readily be deduced directly from sensorgrams and used to standardize the formulation of phage therapies. Furthermore, PLI can be used to detect bacterial contamination since inherent to any phage therapy diagnostic is the ability to detect bacteria. PLI is particularly suited for bacterial testing because it can operate in complex media. Highlighting this capability, we showed that PLI can detect bacterial contamination of baby formula in less than 5 min, and confirmed it was a positive signal from a false-positive signal by observing the lysis signature. This is 200× faster compared to traditional microbiology bacterial enumeration assays, which take 18 h. Bacterial testing is important, as every year 600 million people get sick and 400,000 die due to bacterial food contamination, costing low to middle income countries over $100 billion^59^. Food contamination is also a major concern to high income countries as recently observed from the baby formula crisis in the United States. Ultimately, our vision for PLI is that it will aid in standardizing phage screening that is complementary to current methods, so that phage therapeutic cocktails are formulated rationally rather than at ad hoc, and that PLI can be used to protect against foodborne illness.

## Methods

### Bacteriophages and strains

The bacteriophage strain that was used was T7 bacteriophage. This phage was received from Dr. Kevin Yehl. The bacterial strains of *BW25113* and *BW25113 ΔwaaC* and *ΔtrxA* (“IYB5758”), were received from Dr. Ido Qimron (Tel Aviv University).

### Chemicals and reagents

PBS (ThermoScientific 10× Stock), Tween-20 Ultrapure (ThermoScientific), LB Broth, Miller (Luria–Bertani) (Difco), CsCl (Thermo Fisher Scientific), S-NHS-Biotin (APExBio), Agar Molecular Genetics (Fisher Bioreagents), SYBR™ Safe (ThermoFisher), Liquid Baby Formula Neuropro (Enfagrow), DMSO (Thermo Fisher Scientific), Polyethylene Glycol (PEG) MW: 8000 (MP Bio), Polyethylenimine (PEI) MW: 10,000 (Polysciences, Inc.), Octet ® Streptavidin (SA) Biosensor (Sartorius), Octet ® Amine Reactive 2nd Generation (AR2G) Biosensor (Sartorius), Deionized Autoclaved Water, and Kanamycin (Goldbio).

### Microplate reader assay

An overnight bacterial culture was diluted 1:100 and grown to an OD of 0.7 in a shaking incubator at 37 °C. Then 200 μL of the culture was added to a 96 well plate and T7 phage was added at varying MOIs. The OD600 was then measured every 2 min for 18 h using the Synergy H1 microplate reader.

### Bacterial enumeration assay

Bacterial cultures were serially diluted with sterile PBS in a 96 well plate to a total volume of 100 μL (dilution factor is generally 1:10). A LB-Agar plate was then warmed in an incubator for 30 min. After 30 min, 2 μL of the diluted bacteria solutions were aliquoted onto the plate using a multichannel pipette. The spots were allowed to dry and then placed into a plate incubator (37 °C) overnight. In the morning the plate was imaged using the Bio-Rad ChemiDoc™ Imaging System, and the number of colony-forming units per mL was calculated by counting the number of colonies at the highest dilution, dividing by the volume aliquoted (2 μL), multiplying by a thousand, and then multiplying by the dilution factor.

### Plaque assay

Top-agar was liquefied by microwaving, and then cooled to 55 °C using a dry heat bath. 500 μL of an overnight bacterial culture was added to 5 mL of top-agar (0.6% agar in LB Media), and quickly poured onto antibiotic-free LB-Agar plates. Phage lysates were serially diluted by a factor of 10 to the order of 10^−8^ in a 96-well plate, which was then added to the bacterial lawn (2 μL per dilution). After the spots dried, the plate was placed in the incubator at 37 °C for 2–3 h or overnight at room temperature so that translucent spots can form. PFU per mL was calculated by counting the number of plaques at the highest dilution, dividing by 2, multiplying by 1000, and multiplying by the dilution factor.

### Phage PEG precipitation

T7 phage was added to day cultures of BW25113 (OD ~ 0.7) at an MOI of ~ 0.001, and then placed into a shaking incubator (250 RPMs, 37 °C) for 2 h. After 2 h, the cultures were removed and chloroform was added to a concentration of 0.5% (v/v) and then vigorously shaken. This mixture was left for an hour at room temperature. The phage lysates were then placed into 50 mL falcon tubes and centrifuged at 4000 rpms for 20 min at room temperature. A separation of chloroform and lysate emerges, and the phage lysate is removed (top layer) and filtered using sterile syringe filters (0.2 μm PES from VWR). 5 g of PEG (MW: 8000) and 4.87 g. NaCl was added to each 50 mL tube of phage lysate. This solution was then shaken until all of the solid was dissolved and incubated overnight at 5 °C. After incubation, the solution was spun down at 12.1 k rpm in an Eppendorf fixed angle 50 mL rotor for an hour. A phage pellet sedimented at the bottom of the tubes and the excess solution was discarded to waste. The pellets would then be resuspended in 500 μL of PBS (1×).

### Ultracentrifugation purification

Cesium Chloride was the medium used for ultracentrifugation. For making layered density gradients, 0.7 g/mL, 0.9 g/mL, and 1.1 g/mL CsCl solutions were prepared in AAS buffer (0.1 M. ammonium acetate, 10 mM NaCl, 1 mM CaCl_2_, 5 mM MgCl_2_, pH 7)^26^. SYBR Safe (10,000×) was also added to improve phage visualization. A SW41Ti rotor was used (Beckman) for these experiments with Ultra-Clear ™ Tubes (Beckman 344059). The volume of these tubes is 13.2 mL so 3.3 mL of each density gradient was added layering from the lightest gradient to the heaviest from the bottom of the tube with 1 mL of phage PEG precipitate added to the top. The centrifuge was run at 25,000 rpm (100,000×*g*) for 2 h at 8 °C to establish equilibrium. Once completed, the tubes would be visualized and imaged using an LED transilluminator (Invitrogen dual-led blue-white LED) and smart phone camera, respectively. Samples were collected by puncturing the tube with a needle and drawing up the phage. Image J was used for image analysis. The CsCl was then removed from the phage sample via a 48 h. dialysis cycle in 1× PBS in a dialysis membrane with a molecular weight cut off of 14,000 (Sigma). Plaquing assays were carried out to determine the efficiency of the purification process.

### Phage biotinylation

The S-NHS-Biotin was dissolved in DMSO at a concentration of 34 mM (in 1 mL of DMSO). After cesium chloride (CsCl) density gradient ultracentrifugation and dialysis, 1.83 × 10^11^ PFUs of T7 was mixed with S-NHS-Biotin (APExBIO), volumetrically as 1/50 of total volume, 1 M NaH_2_PO_4_ (pH 7), producing a total volume of 136.3 μL. This was then reacted for 12 h in the dark at room temperature. The resulting biotinylated-phage lysate was then dialyzed to remove excess biotin. Throughout this process at every step of functionalization and purification, the phage activity was examined through plaquing for each step to determine activity.

### Biolayer interferometry (BLI) assay

BLI studies were performed using an Octet ® N1 instrument (Sartorius) at 23 °C with shaking at 2200 RPM (or 37 °C for formula studies). Prior to using Amine Reactive 2nd Generation (AR2G) or Streptavidin (SA) biosensors, they were hydrated in autoclaved nanopure water (18.2 Mohm) for 10 min. Samples and buffer steps were conducted in black 0.6 mL tubes. Volumes in these tubes were 400 μL. The steps of the BLI method were run in a series of these tubes. Tube 1 was PBS-T (0.1%). Tube 2–5 were a series of biotinylated phage samples ranging from 2 × 10^5^ to 3 × 10^8^ PFU/mL. The series was prepared through 1:10 sequential dilutions from the most concentrated sample, 3 × 10^8^ PFU/mL, in 1× PBS. Tubes 3–6 were PBS-T (0.1%) for wash steps. Tube 7 was *BW25113* or *BW25113ΔwaaCΔtrxA* or ECOR strain suspended in PBS after 4 washing cycles. The bacterial samples were a 5 mL overnight culture (OD ~ 2.0) concentrated to 400 μL. Tubes 8–11 are PBS-T (0.1%) for wash steps. Tube 12 was a 400 μL aliquot of Luria–Bertani (LB) Broth from Difco. The BLI method was run as follows. Tube 1 (Equilibration) for 300 s, Tube 2 (Loading) for 3600 s, Tube 3 (Wash 1) for 300 s, Tube 4 (Wash 2) for 300 s, Tube 5 (Wash 3) for 300 s, Tube 6 (Wash 4) for 300 s, Tube 7 (bacterial addition) for 3600 s, Tube 8 (Wash 5) for 300 s, Tube 9 (Wash 6) for 300 s, Tube 10 (Wash 7) for 300 s, Tube 11 (Wash 8) for 300 s, Tube 12 (LB Incubation) for 30,000 s (Overnight). Results were recorded in BLI software via the sensorgram. The raw data was then exported as a .csv file for graphing and interpretation of specific steps.

### Scanning electron microscopy (SEM)

Streptavidin (SA) biosensors (Sartorius) were functionalized with T7-bio (biotinylated bacteriophages) and introduced to live bacteria cells. These biosensors were then prepared for SEM by using a primary fixative (2% paraformaldehyde, 2.5% glutaraldehyde, sodium cacodylate buffer, pH 7.1). The biosensors were submerged in this solution for 45 min at room temperature. Then the biosensors were rinsed with 0.05 sodium cacodylate buffer 3 times, 10 min each time. The biosensors were then dehydrated through a series of ethanol incubations: 25% for 15 min, 50% for 15 min, 75% for 15 min, 95% for 15 min, 100% for 30 min, 100% Microscopy-grade for 30 min, and a final incubation in 100% microscopy grade ethanol prior to critical point drying. Critical Point Drying (CPD) was done using liquid carbon dioxide to completely dehydrate the sample. A thin layer of gold (~ 7 nm) was sputter coated on the sample and the sample was mounted by placing the biosensors on a carbon coated adhesive surface so that the biosensor is orthogonal to the plane of the microscope for surface visualization. The microscope that was used was a Zeiss 35 VP.

### Supplementary Information

Supplementary Information 1.

Supplementary Information 2.

## Data Availability

All data generated or analyzed during this study are included in this published article and its Supplementary Information Files. All bacterial strains and bacteriophages are available upon reasonable request to the corresponding author.
