# Supplementary material for: Phage-layer interferometry: a companion diagnostic for phage therapy and a bacterial testing platform
Source: Sci Rep. 2024 Mar 12;14:6026. doi: 10.1038/s41598-024-55776-1 (PMC10933294; doi:10.1038/s41598-024-55776-1)
Supplement: Supplementary file 1 — Supplementary Information 1. [file 41598_2024_55776_MOESM1_ESM.pdf]

Supplementary Material

Phage-layer interferometry: a companion diagnostic for phage therapy and a bacterial testing platform

Patrick Needham[1], **Richard C. Paige**[1], **Kevin Yehl**[1, *]

[1]Miami University, Department of Chemistry and Biochemistry, Oxford, 45056, USA

[*]yehlk@miamioh.edu

Supplementary Note I:

Objective criteria to distinguish between insensitive strains (i.e., bacteria that do not bind or bacteria that bind but do not lyse), resistant (i.e., bacteria that bind and lyse but continue to grow), and sensitive strains (bacteria that lyse).

Sensitive:

- Fast lysing: Bacteria that have fast binding followed by a sudden decrease in signal due to lysis during the bacterial 'binding step' (overall negative slope during binding).
- Slow lysing: Bacteria that have strong binding (slope > 0.005) and decreasing lysis signal (slope < 0.0001) showing the sigmoidal lysis pattern indicated by a low R2 value (R2 < 0.5).

Insensitive:

- Bacteria that have weak binding (slope < 0.002) and decreasing signal for lysis (negative slope without the sigmoidal lysis pattern, R2 > 0.5).

Resistant:

- Bacteria that have strong binding (slope > 0.002) and increasing signal during the lysis step (positive slope).

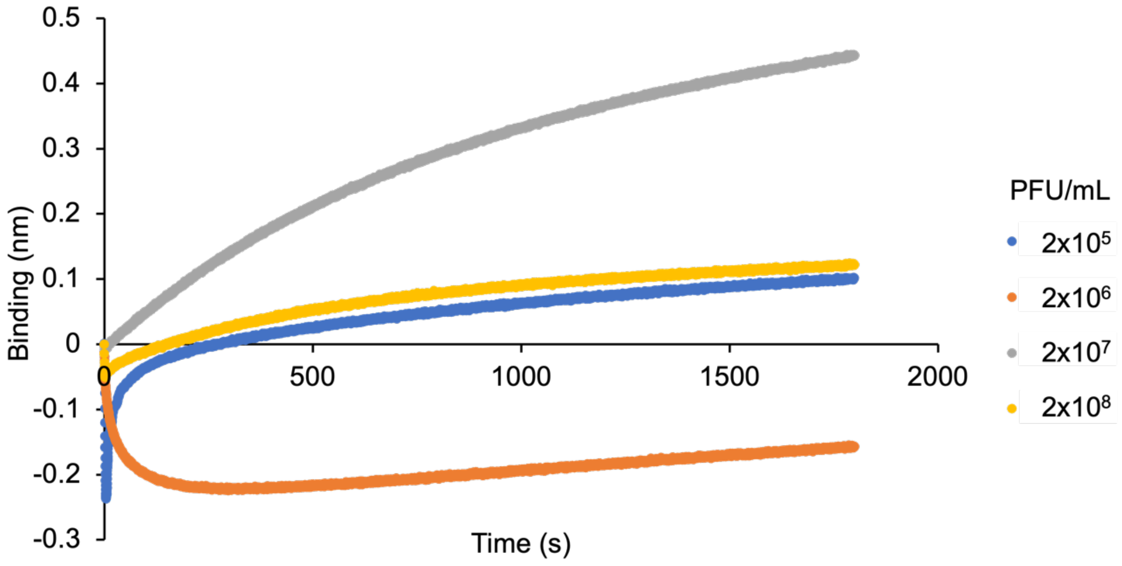

Figure S1. T7-Bio loading onto SA biosensor. SA sensors were incubated in varying concentrations of T7-bio (200 uL).

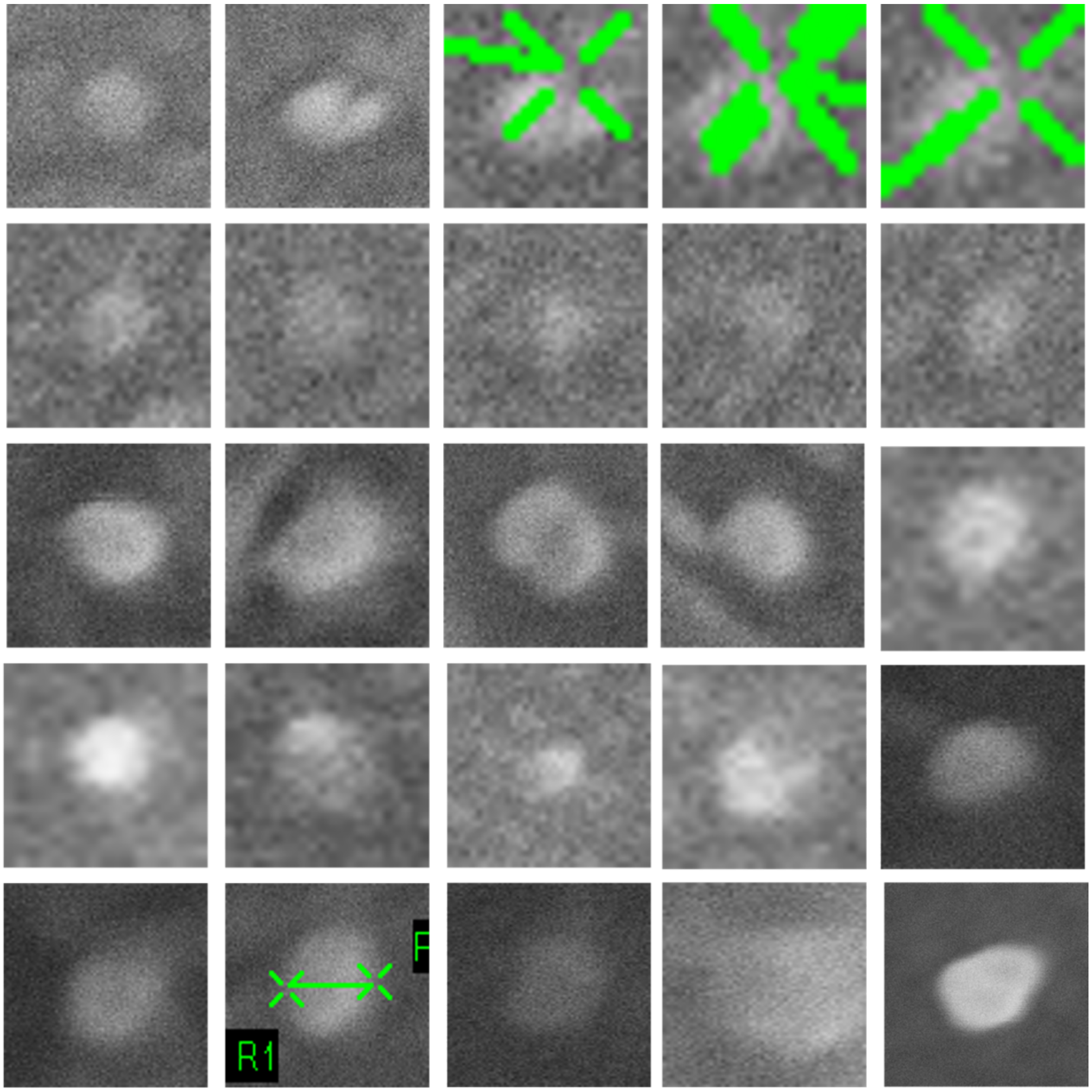

Figure S2. SEM analysis of T7-bio particles on biosensor surface. A collage showing all 25 SEM images of phage particles used for characterizing T7-bio dimensions (Fig. 2d histogram analysis).

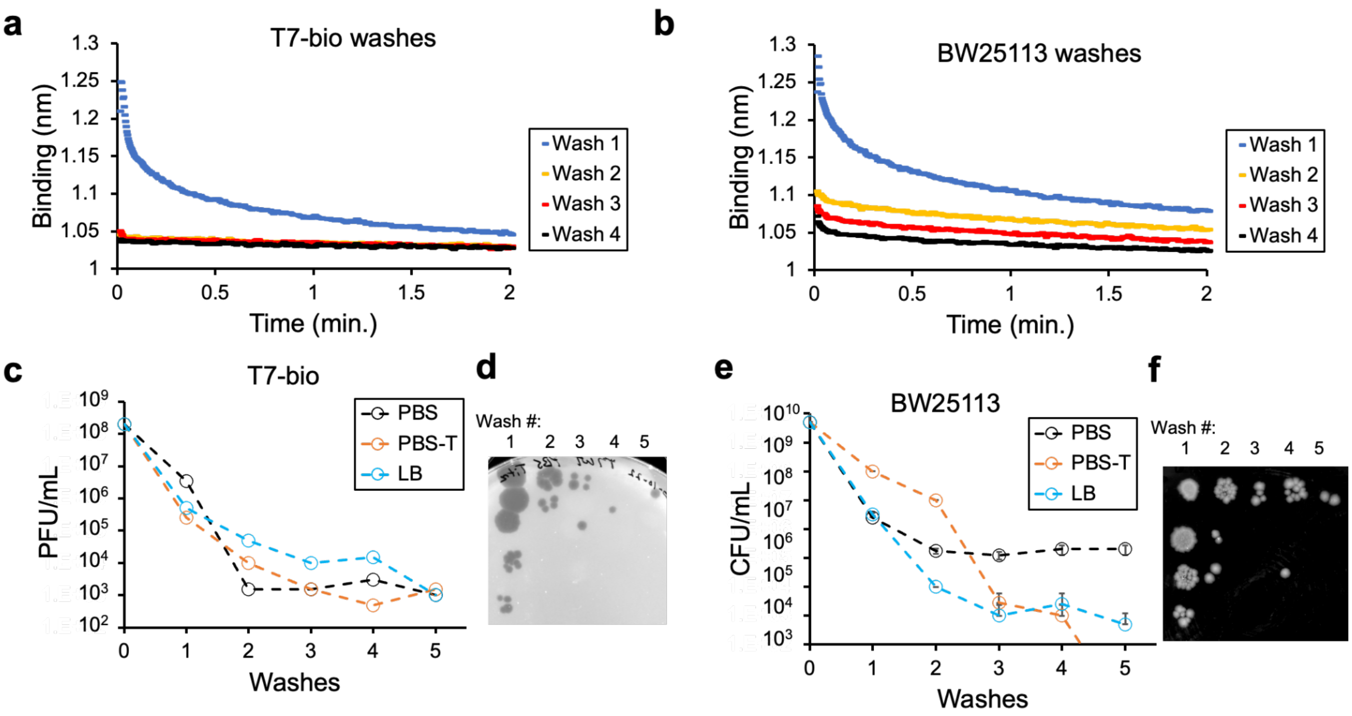

Figure S3. Biosensor washing. (**a**) Overlayed sensorgrams showing the washing steps in PBS tween-20 buffer (PBS-T) after sensor loading with T7-bio. (**b**) Overlayed sensorgrams showing the wash steps in PBS-T after bacterial association. (**c, e**) Plot summarizing the amount of phage (**c**) or bacteria (**e**) washed away from the SA biosensor after each 400 µL wash step in varying buffers (PBS, black; PBS-T, orange; or LB, blue). Points represent the mean of 3 replicates with error bars representing the standard deviation. (**d, f**) Images showing the corresponding plaque (**d**) or bacterial enumeration (**f**) assay used to quantify washing efficacy.

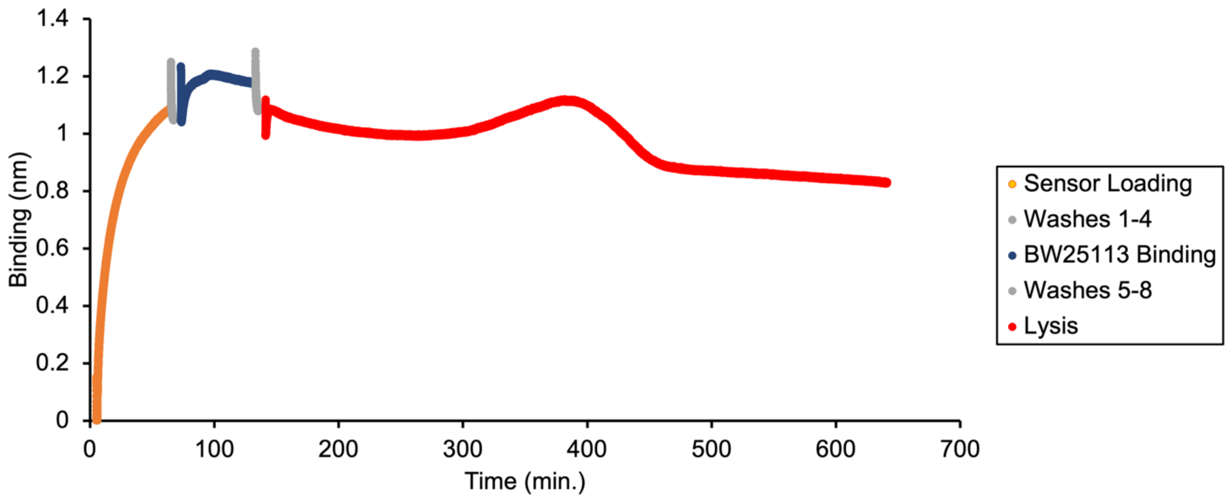

Figure S4. Sensorgram of complete PLI experiment. The sensor loading stage (orange) is the addition of T7-bio to the biosensor surface. Two wash stages were conducted after phage and bacterial association (gray). The bacterial binding step (navy blue) illustrates the binding of *BW25113* to T7-bio on the surface of the biosensor after the first wash stage. The lysis step (red) shows the interactions of T7-bio and *BW25113* in LB media.

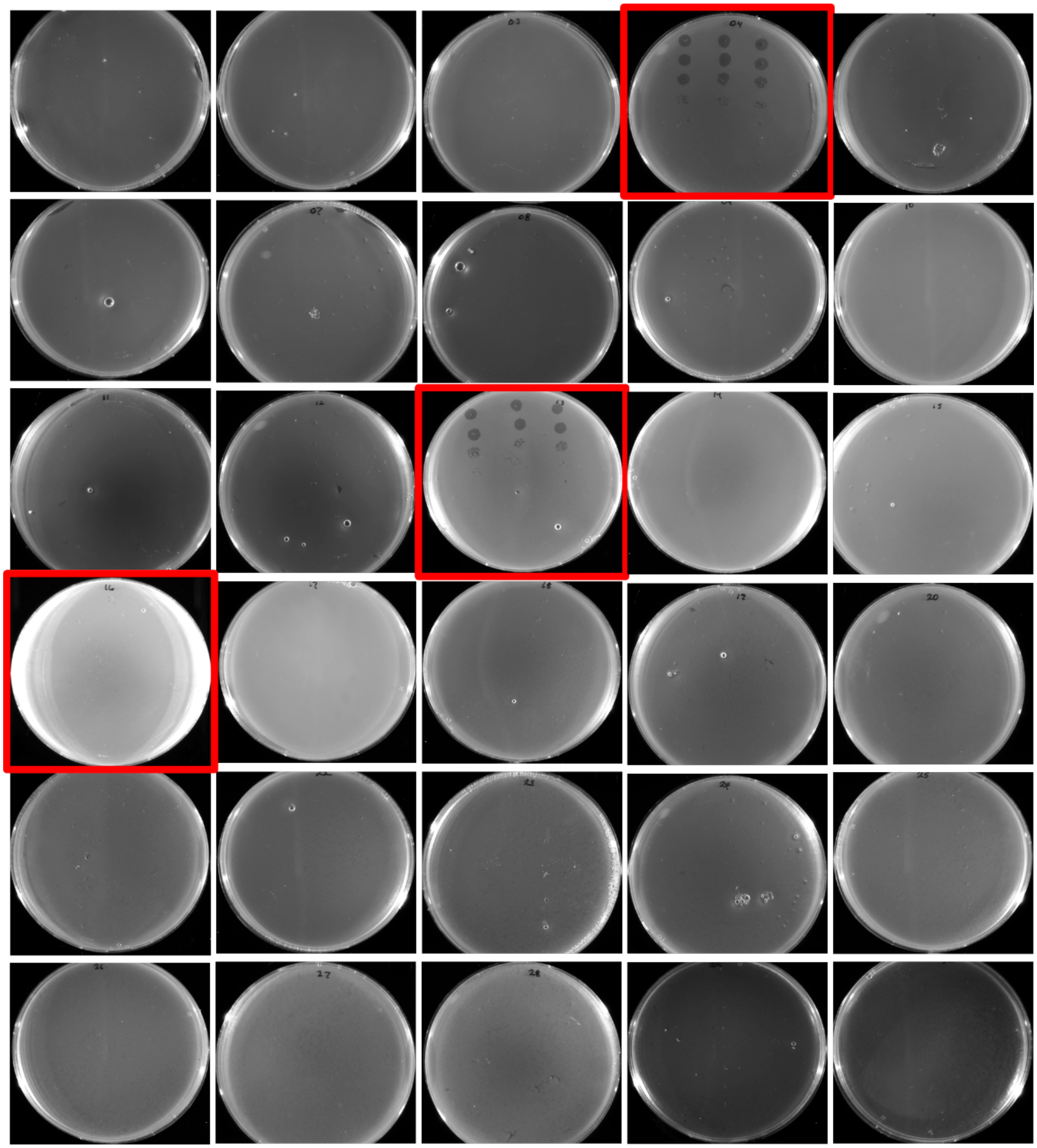

Figure S5. Plaque assays run on the first 30 strains of the ECOR collection. Wild type T7 phage was utilized for these assays. The plate on the top left corner is ECOR01 and increases moving from left to right, with ECOR30 being in the bottom right corner. The red borders on the plates are there to indicate plaque formation.

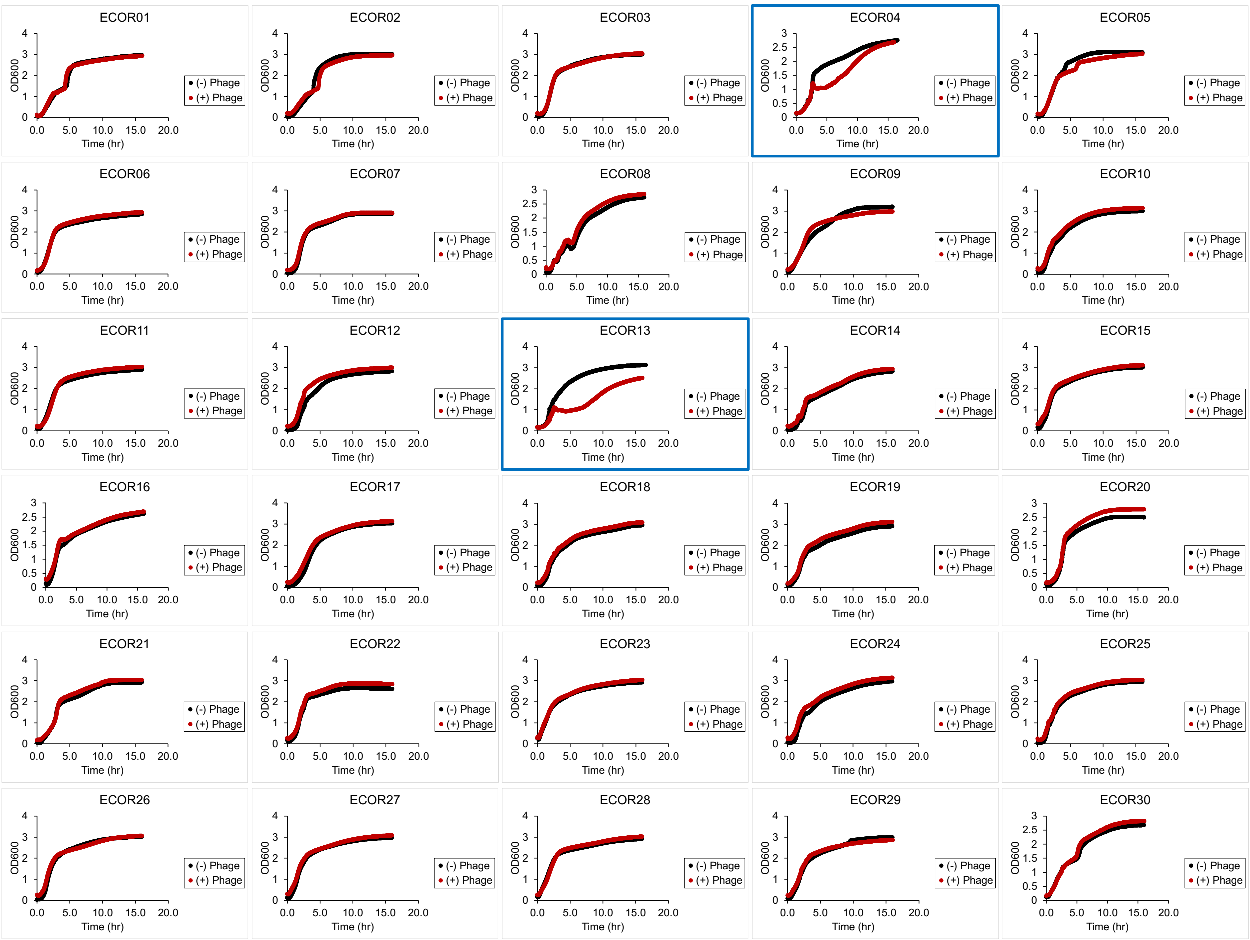

Figure S6. Plate reader kinetic growth assay of first 30 strains of the ECOR collection. The red line is indicative of bacterial strains that were introduced to phage. The black lines are samples only including bacteria. The blue boxes are to illustrate observed phage killing.
